# COVID-19 vaccine refusal associated with health literacy: findings from a population-based survey in Korea

**DOI:** 10.1186/s12889-023-15182-0

**Published:** 2023-02-06

**Authors:** Inmyung Song, Soo Hyun Lee

**Affiliations:** College of Nursing and Health, Kongju National University, 56 Gongjudaehak-Ro, 32588 Gongju-Si, Chungcheongnam-do Republic of Korea

**Keywords:** COVID-19, Vaccine refusal, Vaccine hesitancy, Health literacy, Subjective health status

## Abstract

**Background:**

Poor health literacy is associated with lower utilization of preventable services. However, the relationship between health literacy and COVID-19 vaccine hesitancy remains controvertible.

**Methods:**

This study used data from 229,242 individuals who completed the Community Health Survey in Korea from August 16 to October 31 in 2021. To operationalize COVID-19 vaccine hesitancy, we measured vaccine refusal, which is defined as not having been vaccinated and not intending to get vaccinated against COVID-19. Health literacy is operationalized in two dimensions; the ability to understand spoken directions from health professionals and the ability to understand written information regarding health. Covariates include sex, age, educational level, marital status, employment status, basic living security pension status, and subjective health status. Two multivariable logistic regression models were run to determine factors associated with COVID-19 vaccine refusal. Model 1 included sociodemographic characteristics and subjective health status. Model 2 added two health literacy variables. Odds ratio (OR) and 95% confidence intervals (CI) were calculated.

**Results:**

Only 3.9% of the Korean adult population were estimated to refuse COVID-19 vaccine. The most commonly cited reasons for COVID-19 vaccine refusal were concerns about vaccine adverse events (47.6%), followed by the assessment of one’s own health status (29.5%). Individuals who found spoken directions very difficult to understand were more likely to refuse COVID-19 vaccine than those who found spoken directions very easy (OR = 1.55, 95% CI: 1.28–1.87, *p* < 0.001). People who did not pay attention to written information were more likely to refuse COVID-19 vaccine than those who reported it to be very easy to understand (OR = 1.28, 95% CI: 1.13–1.45, *p* < 0.001). People in all other categories of the literacy spectrum for either spoken or written information did not have an increased risk of COVID-19 vaccine refusal.

**Conclusion:**

Health literacy was significantly associated with COVID-19 vaccine refusal. Health literacy programs could be beneficial to reduce vaccine refusal, particularly for the people who find spoken directions from health professionals very difficult to understand and those who do not pay attention to written information.

## Introduction

According to a survey of a representative sample from 19 countries in June 2020, the proportion of respondents that expressed unwillingness to accept a COVID-19 vaccine ranged from 11.4 to 45.2%, raising concerns among public health experts [1]. Even before the COVID-19 pandemic hit the globe, the World Health Organization (WHO) recognized vaccine hesitancy as one of ten global health threats [2]. Vaccine hesitancy refers to the behaviors of individuals who refuse or delay the acceptance of vaccines that are available for vaccination services [3]. Some regarded vaccine hesitancy as an “ambiguous notion” that encompasses a set of behavior, beliefs, and attitudes towards vaccination [4]. This view mirrors the existence of discrepancies among experts in how to define vaccine hesitancy [5]. More recently, vaccine hesitancy was defined as “a state of indecisiveness regarding a vaccination decision, independently of behavior” [6]. The WHO summarized the reasons for vaccine hesitancy in general as the lack of confidence in vaccines or providers, complacency of not recognizing the need for a vaccine, and inconvenient access to vaccines [7]. A key reason for COVID-19 vaccine hesitancy was concerns about safety, in part because the vaccines were developed and produced at an accelerated speed to quickly respond to the pandemic [7, 8].

Vaccination experts concur that vaccine hesitancy is influenced by individuals’ characteristics such as knowledge, information, and health literacy [9, 10]. Health literacy is defined as “the degree to which individuals can obtain, process, understand, and communicate about health-related information needed to make informed health decisions [11].” Poor health literacy is associated with worse health outcomes and lower use of health services for chronic conditions, such as diabetes and asthma [12, 13]. Inadequate health literacy was also linked to lower use of preventive services, such as influenza and pneumococcal vaccinations [14, 15]. Likewise, willingness to accept COVID-19 vaccine is lower among adults with low health literacy scores and individuals who are less capable of detecting fake news about the vaccine, according to an online survey conducted in France [16]. In contrast, another survey among Italian adults shows that how well one understands information on vaccine is not associated with intention to get vaccinated against COVID-19 [17].

Unlike traditional vaccines, COVID-19 vaccines were developed and approved for use at an unprecedented pace since rapid mass vaccination was considered to be a global security issue [8]. This created many challenges in communicating accurate vaccine information to the general public [18]. While health literacy can play a major role in vaccination intentions, earlier studies were based on online survey data [16, 17, 19]. Therefore, there remains a gap in our understanding of the association between COVID-19 vaccine hesitancy and health literacy. The gap appears to be especially wide in the Korean context. To the best of our knowledge, no general link has been explored between health literacy and COVID-19 vaccine hesitancy in the Korean population. Some researchers only focused their attention on the ability to seek, search, understand, and evaluate health information online, which is referred to as eHealth literacy, as a predictor of intention to vaccinate against COVID-19 [20]. An online survey conducted in South Korea indicates that lower vaccine hesitancy is associated with the use of on- and off-line media to obtain information on the perceived benefits of vaccination [21], which does not represent health literacy per se. Given the paucity of evidence in the Korean context, this study aims to estimate the prevalence of COVID-19 vaccine hesitancy in the Korean adult population aged 19 years and older and to investigate the association between COVID-19 vaccine hesitancy and health literacy using data collected from a sample representative of the population in 2021 when COVID-19 vaccines were widely available.

## Methods

### Data

This study used data from the Community Health Survey (CHS) in Korea that was conducted by the Korea Disease Control and Prevention Agency from August 16 to October 31, 2021. The annual survey that began in 2008 collects data using in-person interviews. In 2021, a total of 229,242 individuals completed the survey [22]. The CHS uses a complex survey design in which administrative areas were cluster sampled using residential type as a stratification variable. A sample of households within each area were selected using systematic sampling. All members aged 19 and older of selected households were interviewed. All participants in the 2021 CHS were used for analysis.

### Measures

For the purpose of this analysis, we examined COVID-19 vaccine refusal, which is an element of the much more comprehensive notion of COVID-19 vaccine hesitancy [4]. COVID-19 vaccine refusal is defined as not having been vaccinated and not intending to get vaccinated against COVID-19. The 2021 CHS included a set of new questions regarding COVID-19 vaccines, allowing for the exploration of the important subject of vaccine hesitancy. Participants were asked to indicate whether they have received a COVID-19 vaccine at the time of the survey. Those who have not been vaccinated were then asked to rate if they intend to get vaccinated against COVID-19 on a four-item scale (strongly agree, agree, disagree, strongly disagree). The respondents who answered ‘disagree’ or ‘strongly disagree’ on the scale were categorized as the vaccine refusal group. The vaccine acceptance group comprised those who have already been vaccinated and those who answered ‘strongly agree’ or ‘agree.’

Health literacy is operationalized in two dimensions; the ability to understand spoken directions from health professionals and the ability to understand written information regarding health. In the 2021 CHS, respondents were asked, “how difficult is it for you to understand spoken directions from health professionals like doctors and nurses?” and “how difficult is it for you to understand health information written on the newspaper, the web, etc.?” To the question regarding spoken directions, the respondents were to select one of the following items: very easy, easy, difficult, and very difficult. To the question regarding written information, the item of “do not pay attention to written information on health” was also provided for selection.

### Covariates

Vaccine hesitancy is shown to be influenced by a number of sociodemographic factors, such as age [23], gender [24–26], educational level [23, 25, 26], marital status [26], income [7, 19, 26, 27], and employment status [7, 24, 27]. Worsening subjective health status also was associated with an increased risk of COVID-19 vaccine hesitancy [27]. Based on the literature review, we included sex, age, educational level, marital status, employment status, basic living security pension status, and subjective health status as covariates in statistical models. Age was grouped into 19–29, 30–39, 40–49, 50–59, 60–69, and ≥ 70 years. Educational level was divided into up to primary education, middle school, high school, and college or higher levels. Marital status was categorized into currently married, ever-married, and never-married. The ever-married category included the divorced and the widowed. Employment status was categorized into salaried, self-employed, and economically inactive. To account for financial situation, respondents were classified into those who received a basic living security pension (i.e., cash assistance for the needy) and those who did not. Subjective health status was measured in five categories (very healthy, healthy, neutral, unhealthy, very unhealthy).

### Statistical analysis

The characteristics of study subjects were described in terms of the number of respondents in the sample, according to sociodemographic, health status, and health literacy variables. By adjusting the frequency with sample weights that were used in the sampling design of the CHS, we calculated the estimated percentage of the population. The χ^2^ (chi-square) test was used to test if COVID-19 vaccine refusal differed among the categories of each variable. Two multivariable logistic regression models were run to determine the association between health literacy and COVID-19 vaccine refusal. Model 1 included sociodemographic characteristics and subjective health status. Model 2 added two health literacy variables. Multivariable-adjusted odds ratio (OR) and 95% confidence intervals (CI) were calculated. We computed *R*^*2*^ of Nagelkerke, as recommended to be used as a measure of effect size for logistic regression models in the literature [28].

All statistical analyses were performed by using SAS version 9.4 (Cary, NC, USA). The Institutional Review Board of Kongju University approved the study protocol and waived the requirement for informed consent (reference No. KNU_IRB_2022-094).

## Results

Only 3.9% of the study population were estimated to refuse COVID-19 vaccine (Table 1). The rest were either already vaccinated or intending to get vaccinated in the future. Spoken directions from health professionals were very easy and somewhat easy for 22.7% and 57.3%, respectively, to understand. Written information was very or somewhat easy for 71.3% to understand, whereas 12.0% did not pay attention to it. The most commonly cited reasons for COVID-19 vaccine refusal were concerns about vaccine adverse events (47.6%), followed by the assessment of one’s own health status (29.5%) (Table 2).

**Table 1:** Characteristics of study subjects (n = 229,242)

Variable	Category	N	% (weighted)
COVID-19 vaccine refusal	Yes	7,872	3.9
	No	221,370	96.1
Sex	Male	104,498	49.6
	Female	124,744	50.4
Age group, years	19–29	24,695	16.6
	30–39	25,733	15.5
	40–49	35,536	18.8
	50–59	43,074	19.7
	60–69	47,528	15.4
	≥ 70	52,676	13.9
Educational level	≤ primary education	51,141	11.8
	Middle school	24,351	7.7
	High school	77,097	36.4
	College or higher	76,519	44.1
Marital status	Currently married	144,107	61.0
	Ever-married	45,504	14.6
	Never-married	39,560	24.4
Employment status	Self-employed	41,056	14.5
	Salaried	92,168	47.6
	Economically inactive	93,021	37.9
Basic living security recipient	Yes	10,806	3.9
	No	218,415	96.1
Subjective health status	Very healthy	14,650	7.6
	Healthy	78,388	37.8
	Neutral	97,386	42.4
	Unhealthy	31,668	10.3
	Very unhealthy	7,144	1.9
Spoken directions from health professionals	Very easy	49,895	22.7
	Somewhat easy	126,920	57.3
	Somewhat difficult	46,366	18.4
	Very difficult	5,880	1.7
Written information	Very easy	40,627	20.0
	Somewhat easy	107,516	51.3
	Somewhat difficult	36,462	14.7
	Very difficult	6,961	2.0
	Do not pay attention	37,571	12.0

N is the frequency in the sample. % (weighted) is the estimated percentage in the population

**Table 2:** Reasons cited for COVID-19 vaccine refusal

Reason	No. of respondents	%
Concerns about and past experience of vaccine adverse events	3,747	47.6%
One’s own health status	2,323	29.5%
Not trusting the government’s immunization policy	482	6.1%
Lack of vaccine efficacy	413	5.2%
Not trusting the safety management of pharma companies and vaccination facilities	304	3.9%
Others	603	7.7%
Total	7872	100.0%

The prevalence of COVID-19 vaccine refusal differed by all of the sociodemographic, health status, and health literacy variables examined in this study except for sex (*p* < 0.001, Table 3). COVID-19 vaccine refusal was reported in 7.2% of people for whom spoken directions were very difficult and 3.5% of people for whom spoken directions were very easy. 4.5% of people who reported written information to be very difficult to understand and 4.1% of those who did not pay attention to written information were vaccine-hesitant.

**Table 3:** Prevalence of COVID-19 vaccine refusal vs. acceptance by sociodemographic, subjective health, and health literacy status in 2021

		Refusal		Acceptance		*p*-value
Variable	Category	n	%	n	%	
Sex	Male	3,643	3.5	100,855	96.5	0.499
	Female	4,229	3.4	120,515	96.6	
Age group, years	19–29	1,586	6.4	23,109	93.6	<0.001
	30–39	1,699	6.6	24,034	93.4	
	40–49	1,185	3.3	34,351	96.7	
	50–59	862	2.0	42,212	98.0	
	60–69	947	2.0	46,581	98.0	
	≥ 70	1,593	3.0	51,083	97.0	
Educational level	≤ Primary education	1,533	3.0	49,608	97.0	<0.001
	Middle school	543	2.2	23,808	97.8	
	High school	2,789	3.6	74,308	96.4	
	College or higher	3,001	3.9	73,518	96.1	
Marital status	Currently married	3,893	2.7	140,214	97.3	<0.001
	Ever-married	1,594	3.5	43,910	96.5	
	Never-married	2,379	6.0	37,181	94.0	
Employment status	Self-employed	1,061	2.6	39,995	97.4	<0.001
	Salaried	2,430	2.6	89,738	97.4	
	Economically inactive	4,260	4.6	88,761	95.4	
Basic living security recipient	Yes	678	6.3	10,128	93.7	<0.001
	No	7,193	3.3	211,222	96.7	
Subjective health status	Very healthy	682	4.7	13,968	95.3	<0.001
	Healthy	2,256	2.9	76,132	97.1	
	Neutral	2,796	2.9	94,590	97.1	
	Unhealthy	1,474	4.7	30,194	95.3	
	Very unhealthy	664	9.3	6,480	90.7	
Spoken directions from health professionals	Very easy	1,736	3.5	48,159	96.5	<0.001
	Somewhat easy	4,075	3.2	122,845	96.8	
	Somewhat difficult	1,625	3.5	44,741	96.5	
	Very difficult	422	7.2	5,458	92.8	
Written information	Very easy	1,470	3.6	39,157	96.4	<0.001
	Somewhat easy	3,392	3.2	104,124	96.8	
	Somewhat difficult	1,136	3.1	35,326	96.9	
	Very difficult	314	4.5	6,647	95.5	
	Do not pay attention	1,551	4.1	36,020	95.9	

*p*-values were obtained by using the χ^2^ test

In model 1, people aged 40 years and older were less likely than those aged 19–29 years to refuse COVID-19 vaccine (Table 4, *p* < 0.001). The risk of COVID-19 vaccine refusal was smaller for the self-employed and the salaried than for economically inactive individuals (*p* < 0.001). Unhealthy and very unhealthy individuals were at greater risk of COVID-19 vaccine refusal than very healthy individuals (OR = 1.62, 95% CI: 1.42–1.83; OR = 3.35, 95% CI: 2.84–3.94, respectively, *p* < 0.001 in model 2). People who found spoken directions very difficult to understand were more likely to refuse COVID-19 vaccine than people who found spoken directions very easy (OR = 1.55, 95% CI: 1.28–1.87, *p* < 0.001 in model 2). People who did not pay attention to written information were more likely to refuse vaccine than people who reported it to be very easy to understand (OR = 1.28, 95% CI: 1.13–1.45, *p* < 0.001 in model 2). Model 2 shows an improved effect size compared to model 1, according to Nagelkerke’s *R*^*2*^.

**Table 4:** Odds of COVID-19 vaccine refusal in 2021

			Model 1				Model 2		
Variable (reference)	Category	OR	95% CI		*p*-value	OR	95% CI		*p*-value
Sex (male)	Female	0.92	0.87, 0.97		< 0.01	0.92	0.87, 0.98		< 0.01
Age group, years (19–29)	30–39	1.18	1.05, 1.33		< 0.01	1.18	1.05, 1.32		< 0.01
	40–49	0.53	0.47, 0.61		< 0.001	0.54	0.47, 0.62		< 0.001
	50–59	0.26	0.22, 0.31		< 0.001	0.26	0.22, 0.31		< 0.001
	60–69	0.24	0.20, 0.28		< 0.001	0.24	0.20, 0.29		< 0.001
	≥ 70	0.24	0.20, 0.28		< 0.001	0.23	0.19, 0.27		< 0.001
Educational level (≤ primary)	Middle school	0.88	0.77, 1.02		0.090	0.95	0.82, 1.10		0.507
	High school	0.99	0.87, 1.13		0.873	1.10	0.96, 1.26		0.178
	College or higher	0.93	0.81, 1.07		0.337	1.05	0.91, 1.21		0.522
Marital status (currently married)	Ever-married	1.22	1.12, 1.34		< 0.001	1.20	1.09, 1.32		< 0.001
	Never-married	1.05	0.94, 1.17		0.366	1.04	0.93, 1.16		0.489
Employment status (economically inactive)	Self-employed	0.79	0.71, 0.87		< 0.001	0.79	0.71, 0.87		< 0.001
	Salaried	0.52	0.48, 0.56		< 0.001	0.52	0.48, 0.56		< 0.001
Basic living security (no)	Yes	1.47	1.29, 1.67		< 0.001	1.45	1.28, 1.65		< 0.001
Subjective health status (very healthy)	Healthy	0.76	0.69, 0.85		< 0.001	0.77	0.69, 0.86		< 0.001
	Neutral	0.87	0.78, 0.97		< 0.05	0.87	0.78, 0.97		< 0.05
	Unhealthy	1.62	1.42, 1.83		< 0.001	1.59	1.40, 1.81		< 0.001
	Very unhealthy	3.35	2.84, 3.94		< 0.001	3.10	2.63, 3.66		< 0.001
Spoken directions from health professionals	Somewhat easy					0.95	0.86, 1.04		0.232
(very easy)	Somewhat difficult					1.01	0.90, 1.14		0.849
	Very difficult					1.55	1.28, 1.87		< 0.001
Written information	Somewhat easy					1.00	0.90, 1.10		0.936
(very easy)	Somewhat difficult					1.00	0.88, 1.13		0.958
	Very difficult					1.14	0.93, 1.39		0.212
	Do not pay attention					1.28	1.13, 1.45		< 0.001
Observations		228,978				228,721			
Nagelkerke’s *R*^2^		0.0648				0.0666			
-2 Log L		13,547,591				13,510,691			

OR, odds ratio; CI, confidence intervals. ORs are multi-variable adjusted ORs

## Discussion

In a cross-national survey conducted in June 2020, 20.2% of participants in Korea expressed unwillingness to accept a COVID-19 vaccine if it is available [1]. The sentiment did not seem to improve with the passage of time; in another survey conducted between October to December of 2020, 39.8% of Korean adults were still hesitant or refused to be vaccinated [27]. Even in January 2021, COVID-19 vaccine hesitancy was reported to be considerably high according to online surveys in Korea [21] and China (53.3% and 35.5%, respectively) [29]. However, in the current analysis of data collected between August and October 2021, the prevalence of COVID-19 vaccine hesitancy is estimated to be low at 3.9% in the Korean adult population. This is on par with the prevalence of COVID-19 vaccine hesitancy (5.2%) among a convenience sample of US adults in February 2021 [30]. Such a swift change in vaccination intentions may have been due to the availability of COVID-19 vaccines, clinical trial data on safety and efficacy, and vaccination campaigns [19, 31]. Furthermore, a number of measures that the Korean government implemented may have played a role in increasing the vaccination coverage and intentions [32]. For example, mobile vaccination teams were in operation to remove physical barriers for the disabled. In addition, the government introduced various vaccination incentives, such as easing the requirements of social distancing and 2-weeks self-quarantine on return from travelling abroad for the fully vaccinated.

In the present study, COVID-19 vaccine-hesitant individuals cited concerns about vaccine safety as the most common reason for hesitancy, which was consistent with the previous findings [7, 27]. However, contrary to the earlier study [7], a lack of confidence in vaccine efficacy was rarely cited as the reason for hesitancy. This difference may have occurred in part because this present study used data collected in late 2021 when clinical trial data were available [31], and in another part because safety was a greater concern than a lack of efficacy as a reason for COVID-19 vaccine hesitancy [24].

An individual’s confidence in a vaccine is likely to be associated with ability to understand health information. The COVID-19 vaccine’s rapid pace of development may have led to a proliferation of misinformation, which could have posed a challenge to people with inadequate health literacy [26]. As it happens, concerns about COVID-19 vaccine safety were greater among people with lower health literacy, according to an email survey of US adults in January 2021 shortly after the emergency use authorizations of COVID-19 vaccines [19]. The risk of vaccine hesitancy is greater for people who found obtaining health information a frustrating task than for those who could easily navigate health information [30]. People who relied on social media for information and were susceptible to vaccine conspiracies appear to be hesitant to be vaccinated against COVID-19 [26].

This current study shows that poor health literacy is significantly associated with COVID-19 vaccine hesitancy. However, only the individuals who find spoken direction from health professionals very difficult to understand or those who do not pay attention to written health information have an increased risk of COVID-19 vaccine hesitancy compared to individuals who find health information, spoken and written, very easy to understand. People in all other categories of the literacy spectrum for either spoken or written information did not have an increased risk of vaccine hesitancy. These findings suggest that any incremental efforts to help people with poorest health literacy can improve vaccination intention for some vaccines. Likewise, efforts to increase the reach of written health information could have a material impact on mitigating vaccine hesitancy.

In addition to health literacy, sociodemographic variables, such as sex, age, and employment status, were significant predictors of COVID-19 vaccination hesitancy, which is consistent with the existing literature [23, 25, 27]. Health status was another significant predictor of COVID-19 vaccine hesitancy in the current study. People who rated themselves to be unhealthy were more likely to be COVID-19 vaccine-hesitant than those who perceived themselves to be very healthy. Moreover, assessment of one’s own health status was the second most commonly cited reason for vaccine hesitancy. However, evidence on the impact of health status on vaccine hesitancy is mixed in the literature. While US adults with five or more preexisting conditions were less COVID-19 vaccine-hesitant than people with none [26], German adults with chronic diseases were no less hesitant than those without one [25].

The association between health literacy and COVID-19 vaccine hesitancy shown in this study suggests a need for developing health literacy programs for the general public. A study in Germany showed that while communicating the benefits of COVID-19 vaccines was effective in significantly reducing vaccine hesitancy, its effect in areas with low health literacy was weaker [33]. This implies that increasing general health literacy through preemptive programs could improve the effect of any future urgent vaccination campaigns.

Aside from the general public, health professionals are also at risk of vaccine hesitancy [34]. Healthcare professionals were often the most trusted source of guidance regarding COVID-19 vaccines [35]. For that reason, health professionals’ own vaccine hesitancy could influence the general public’s vaccine hesitancy. There is evidence that some health professionals in the United States had COVID-19 vaccine hesitancy mainly due to safety concerns [36], and that health professionals’ own confidence in a particular vaccine influenced their recommendation to others [37]. Research reveals that a substantial proportion of primary care physicians in the Western hemisphere have inaccurate knowledge regarding vaccines [38]. Unsurprisingly, the literature points to the importance of educating health professionals as well as the general public using various communication tools, in order to reduce vaccine hesitancy [34].

To the best of our knowledge, this study is the first one to document the association between health literacy and COVID-19 vaccine hesitancy by using a large representative sample of the Korean population. This study indicates that only the individuals with lowest health literacy are at increased risk of vaccine hesitancy. These individuals find spoken directions from health professional very difficult to understand and they do not pay attention to written information. Any incremental efforts to help those individuals could reduce COVID-19 vaccine hesitancy. Despite the strengths, the findings should be interpreted with caution due to the following limitations. First, this study showed only association not causality due to the nature of a cross-sectional study design. Second, the measures of health literacy may not have fully captured the varied dimensions of a complex concept of health literacy, which has been measured by using a number of items [25]. Third, this study used self-reported data from the survey and therefore was subject to recall bias.

## Conclusion

Health literacy is significantly associated with COVID-19 vaccine refusal. At most risk of COVID-19 vaccine refusal are people who have the most difficulty understanding directions from health professionals as well as those who do not pay attention to written health information. Any incremental efforts to help people understand spoken directions and pay attention to written health information will be beneficial to reduce COVID-19 vaccine hesitancy.

## Data Availability

The datasets generated and/or analysed during the current study are available in the Korea Disease Control and Prevention Agency repository (https://chs.kdca.go.kr/chs/rdr/rdrInfoDownMain.do).
